# Achieving Integrated Care for Older People: What Kind of Ship? Comment on "Achieving Integrated Care for Older People: Shuffling the Deckchairs or Making the System Watertight for the Future?"

**DOI:** 10.15171/ijhpm.2018.44

**Published:** 2018-05-07

**Authors:** Rod Sheaff

**Affiliations:** University of Plymouth, Plymouth, UK.

**Keywords:** Integrated Care, Primary Care, Multi-morbidity, Chronic Illness, Organisational Integration, Systematic Review

## Abstract

This paper considers an implication of the idea that proposals for integrated care for older people should start from a focus on the patient, consider co-production solutions to the problems of care fragmentation, and be at a system-wide, cross-organisational level. It follows that the analysis, design and therefore evaluation of integrated care projects should be based upon the journeys which older patients with multiple chronic conditions usually have to make from professional to professional and service to service. A systematic realistic review of recent research on integrated care projects identified a number of key mechanisms for care integration, including multidisciplinary care teams, care planning, suitable IT support and changes to organisational culture, besides other activities and contexts which assist care ‘integration.’ Those findings suggest that bringing the diverse services that older people with multiple chronic conditions need into a single organisation would remove many of the inter-organisational boundaries that impede care ‘integration’ and make it easier to address the interprofessional and inter-service boundaries.

Gill Harvey and her co-authors^1^ begin their editorial uncontroversially by stating what problems ‘Integrated Care’ addresses: growing populations of older people with multiple chronic conditions need to obtain care from multiple providers, whether concurrently (eg, from general practitioners [GPs] and domiciliary services), sequentially (eg, from primary care to hospital and back) or both. These people often experience frequent, sometimes chaotic, transitions between services or ‘fall through the gaps’ of a fragmented care system. Harvey et al cite some of the evidence for that.

An obvious response to fragmentation is to demand ‘integration,’ and many studies have. As Harvey et al say, the empirical studies report various practical approaches to improving ‘integration,’ obstacles to replicating the successful ones more widely, and the gaps between what patients want from care and what clinicians and health managers prioritise. So Harvey et al recommend three main foci for future care integration projects:

1. Care ‘integration,’ and therefore attempts to improve it, involve system-wide cross-organisational care pathways. Single-organisation or single-sector ‘solutions’ just ‘shuffle the deckchairs.’
2. Start from a focus on the patient.
3. Consider co-production solutions to the above problems.

## Co-production and a Focus on the Patient

Responding in reverse order, ‘patient-centred’ perspectives such as Harvey et al recommend imply that by default arrangement, a patient be her own care-coordinator. This does not mean leaving the patient to fend for herself. Rather, the formal care coordinator acts as her agent, but the patient co-ordinates and co-produces her own care so far as her individual circumstances allow. For both patient and practitioners that requires specific mindsets and skills,^2^ which may have to be learnt. However, the combination of morbidities and their progression (eg, failing memory, loss of mobility) constrain whether a patient is physically and cognitively capable of being her own care coordinator and co-producer. Then an informal carer may have to fulfil the role. Even when patients are capable, ‘gatekeeping’ rules define who can refer whom to what services. In most health systems, many services can only be accessed by referral from a health professional, typically the GP or equivalent. Informal carers acting on the patient’s behalf also face this constraint.

When these circumstances compel practitioners to act as the patient’s care coordinator, they face as Harvey et al say a potential discursive gap between them and the patient or her informal carer. One such gap concerns the patient’s understanding of what she needs to do, and not do, in order to get coordinated care. For instance when one American accountable care organization (ACO) set up care coordination systems, some patients by-passed them by self-referring to duplicate services at the same time.^3^ Perhaps thinking of such circumstances, the National Health Service (NHS) clinicians and managers who advised our recent systematic review^4^ on care coordination spoke of ‘educating’ patients. Another possible (and not incompatible) approach at practitioner level is some form of ‘narrative medicine’ ie, the competence of eliciting *from the patient* her current health status^2,5,6^ and its practical consequences for her; and then basing her care or treatment plan, and the consequent service selection, referrals and coordination, upon that.

## Why Might Integrated Care Help?

A patient-centred view of care ‘integration’ and the system-wide view which Harvey et al recommend imply that the analysis, design and evaluation of integrated care projects should start from the journey which older patients with multiple chronic conditions usually have to make from professional to professional and service to service. That is, from an analysis of system-wide mechanisms through which care coordination between care providers is in fact accomplished, or fails. That involves understanding what contexts they work well in, or not, and for whom.

For most health systems a generic but still empirical overview of these care pathways would be:

1. An illness, accident or an adverse lift-event prompts the patient or their carer go to a ‘first contact’^7^ service: for instance a primary care doctor, ambulance or hospital emergency department (ED).
2. A filtering (eg, formal diagnosis, risk assessment) of the patients with chronic multiple morbidities…
3. …who are then allocated a care coordinator. In most health systems that role falls to some combination of:
(*a*) Patient herself or an informal carer,
(*b*) Primary care doctor (eg, GP, polyclinic doctor), who is often the first contact anyway,
(*c*) Non-medical practitioner (eg, nurse, therapist, psychologist, social-worker),
(*d*) Hospital outpatient (ambulatory) service.
The care coordinator reviews the patient’s needs and at need refers her to other professions, services or organisations for some combination of diagnostic tests or reports, other primary care services (maybe provided by a multidisciplinary team [MDT]), or hospital care. In some cases the coordinator formulates, even documents, an explicit care plan for the individual patient.
Provision of these additional services and any further care by the ‘first contact’ service.
Reporting and/or referral back to the care coordinator, after which…
…steps 4-6 repeat until the patient is discharged or dies.

Each step or link in this network of transitions establishes, maintains or disrupts cross-sectional, longitudinal, flexible, access, informational and relational continuity of care for the patient. As Harvey et al say, which links even exist, and which of them enable continuity of care and care coordination, varies by locality and health system.

## System-Wide Cross-Organisational Care Pathways

Accordingly one can classify and evaluate ‘integrated’ care interventions according to which of the above links, hence continuities of care, they address; and so operationalise one of Harvey and colleagues’ recommendations. Our own review^4^ found that recent (2014-2016) research in the Organisation for Economic Co-operation and Development (OECD) countries has focused on ‘integrating’ (linking) separate provider-organisations mostly through the following mechanisms:

1. Referral network management: setting up a district or regional-level body to coordinate the relevant providers using, above all, the following four mechanisms. In the United States, one motive for providers to participate in ACOs was to get their patients access to a wider range of services.^8,9^
2. MDTs, both within and across organisations, emerged as the most important single care coordination mechanism. MDTs were associated with the development of planned referral networks (inter-organisational care pathways),^10^ the development of care planning for individual patients(11 studies)and of demand management systems to filter patient referrals to secondary care.^11,12^ An important contributor to these outcomes was the development of new or expanded boundary spanning roles (for instance, for care coordinators).^13-17^
3. Culture change among the different organisations and (especially) professions working in primary care figures as an independent variable in many studies. (Wholey^18^ however replies that the tasks it must perform, not culture, are the logical starting point for MDT design.) Together, such studies credited culture change as stimulating the development of inter-professional working,^13,19^ greater use of preventive healthcare,^20^ and improved patient experience of care.^21^
4. Health IT systems supported the development of MDTs,^19^ demand management systems, care planning for individual patients and the use of preventive healthcare. Some studies^22,23^ also suggested that IT reduced the costs of healthcare, but partly through automating administrative work rather than influencing service use.
5. Patient-level care planning (ie, the agreement of one overall care plan for the patient, not one separate plan per profession) stimulated preventive care, helped divert patients from hospital to primary care (many studies). A few studies^16,22,24^ found that care planning improved patient experience of care, especially (and corroborating Harvey et al) if the patient was involved.

In summary, these mechanisms in combination led in favourable contexts to greater use of preventive healthcare, fewer hospital referrals, hence improved patient experience and quality of care. Evidence that they reduced the overall costs of healthcare was sparse but did come from several countries (Germany, Switzerland, USA). In such an integrated care setting, patients and carers might be active members of the MDT,^25^ a designated care coordinator or case manager would periodically review and adjust the support they received, and different providers would keep each other informed about changes in the patient’s circumstances and support. Contexts that helped implementing these mechanisms across organisations included previous experience of collaboration,^10,26^ IT systems that accommodated clinicians’ existing working practices,^4^ and workplace cultures that valued other professions’ contributions to care.^13,27^

These findings concerned mainly ‘horizontal’ coordination between primary medical care and other primary care services such as community nursing, the therapies, psychology, pharmacy and social work. ‘Vertical’ integration between primary and hospital care may require different mechanisms and have different effects.

## Organisational Fragmentation and Care Integration

Harvey et al therefore argue that single-organisation or even single-sector ‘solutions’ to care integration just ‘shuffle the deckchairs.’ They observe that many integrated care projects assert the importance of having ‘a strong primary care foundation.’ The problem, however, is that ‘primary care’ is so often equated only with ‘general medical practice’ or the local equivalent. In many health systems the two most important providers of services for older people ie, general medical practice and community health (nursing, therapy) services remain in separate organisations under different ownership. Thus one study^28^ found that 48% of the American ACOs did not include postacute care (whose role is partly similar to that of community health services in NHS-like systems). The other 52% were more likely to have programmes to reduce preventable hospital admissions and for end-of-life care.Mental health services and social care are often separate organisations again. In 2014, for example, 58% of American ACOs did not include mental healthcare providers.^9^ American ACOs are by no means the most fragmented cases.

Recent research already identifies structures and contexts which apparently improve care coordination, continuity, and patient experience. They include:

- Developing cross-profession, cross-service MDTs. A favourable context for this is a history of earlier collaboration (many studies), for each profession to have informal contact, and familiarise itself, with other professions’ roles; which promotes inter-professional trust.^29^ Co-locating staff helps.^14,30^
- Structures and roles – not least, that of care coordinator – which span occupational and organisational boundaries.^15^
- Making separate services’ working practices mutually consistent, for example through an agreed division of labour to reduce role overlap and ambiguity (‘care compacts’)^10^; referral rules; formularies; and uniform, cross-disciplinary training about IT and care coordination.^31^
- Hence, shared workplace culture and climate.
- For larger care groups, planning unified care or referral pathways across the network of providers as a whole, with agreed task allocation and referral rules.^28^
- Sharing resources, linking to missing services such as ‘social prescribing’ and involving the ‘third sector.’^16^
- Data-sharing between providers and structured communication within MDTs, hence compatible and interoperable IT systems, particularly electronic health records^30^; but if they are not usable and useful for clinicians’ everyday working practice, their effect can be counter-productive.
- Alignment of payments and other incentives across different services.^32^

All these would seem easier to implement within a single organisation.

So Harvey et al are right to dismiss single-organisation or single-sector ‘solutions’ to care integration *only* if we assume that these organisations remain as narrow in scope as most primary care providers are in many health systems (eg, Australia, France, Germany, the Netherlands, New Zealand, UK, USA). There, primary care medicine is still mostly provided by small, independent general practices, or even single-handed ‘free professionals,’ which provide few of the other services that older people with multi-morbidity need. No wonder that care for people with multiple long-term chronic conditions is then fragmented and hard to ‘integrate.’ However it is entirely conceivable that a single organisation might combine primary medical care, community and mental health services, and perhaps social care too, within a single organisation. Organisational integration would remove many of the inter-organisational boundaries that impede care ‘integration’ and make it easier to address the inter-professional and inter-service boundaries. Indeed, it is not just conceivable. Horizontal integration of this kind already exists in (eg,) Sweden and Finland, with experiments^33^ in extending it further, and to varying extents elsewhere. Doubtless these systems still have scope for improving care coordination further, but they start with a big structural advantage and give proof-of-concept that organisational integration is feasible. In that sense, to re-cycle one of Gill Harvey’s maritime metaphors, the problem indeed is ‘Whether the ship is actually big enough.’ That means, broad enough.

## Acknowledgements

The systematic review on which this commentary draws was funded by the National Institute for Health Research (NIHR) Health Services and Delivery Research (HS&DR) Programme, and supported by the NIHR Collaboration for Leadership in Applied Health Research and Care South West Peninsula (PenCLAHRC). Besides the present author it was conducted by Sarah Brand, Helen Lloyd, Amanda Wanner, Mauro Fornasiero, Simon Briscoe, Jose Valderas, Richard Byng, and Mark Pearson. The views and opinions expressed above are those of the present author alone, not necessarily those of the HS&DR Programme, NIHR, NHS, the Department of Health and Social Care or his co-researchers.

## Ethical issues

Not applicable.

## Competing interests

Author declares that he has no competing interests.

## Author’s contribution

RS is the single author of the paper.
